# Acceptability and adherence of Balanced Energy Protein (BEP) supplementation among pregnant women in Addis Ababa, Ethiopia: A Formative Study

**DOI:** 10.1371/journal.pone.0344089

**Published:** 2026-03-06

**Authors:** Tigest Shifraw, Hanna Yemane Berhane, Sitota Tsegaye, Hanna Gulema, Hana Sime, Yoseph Yemane Berhane, Workagegnhu Tarekegn, Janaína Calu Costa, Dongqing Wang, Wafaie W. Fawzi, Yemane Berhane

**Affiliations:** 1 Reproductive health and population department, Addis Continental Institute of Public Health, Addis Ababa, Ethiopia; 2 Nutrition and Behavioral Science Department, Addis Continental Institute of Public Health, Addis Ababa, Ethiopia; 3 Global Health and Health Policy Department, Addis Continental Institute of Public Health, Addis Ababa, Ethiopia; 4 Epidemiology and Biostatistics Department, Addis Continental Institute of Public Health, Addis Ababa, Ethiopia; 5 Department of Global Health and Population, Harvard T.H. Chan School of Public Health, Boston, United States of America; 6 Department of Epidemiology, School of Public Health, University of São Paulo, São Paulo, SP, Brazil; 7 Department of Global and Community Health, College of Public Health, George Mason University, Fairfax, Virginia, United States of America; VITAL Pakistan Trust, PAKISTAN

## Abstract

**Background:**

Balanced energy protein (BEP) supplements during pregnancy have shown promise in reducing small-for-gestational-age births and low birth weight in low- and middle-income countries, but practical gaps remain in implementing this supplementation. This formative study assessed the acceptability, adherence, and sharing practices of a ready-to-use BEP supplement among pregnant women in Addis Ababa, Ethiopia.

**Subjects and method:**

From January to February 2023, 45 pregnant women were purposively selected from nine public health centers and provided a 4-week BEP supply with usage guidance. Thirty-nine participants completed in-depth interviews. The framework analysis focused on acceptability, adherence, and sharing. Adherence was calculated as the proportion of sachets consumed or returned empty; partially used or unopened sachets were considered unconsumed.

**Results:**

Acceptability was high, with positive feedback on texture (32/39, 82.1%), smell (35/39, 89.7%), color (38/39, 97.4%), and ease of consumption (33/39, 84.6%). However, 23/39 participants (59%) found the taste overly sweet, which occasionally led to nausea or reduced intake. Over the one month, supplement consumption ranged from 0 to 30 sachets, with a mean intake of 21.9 (SD = 9.5). High adherence (≥80% consumption) was observed in 25/39 (64.1%) of participants. Intake was facilitated by provider counseling and family support, while fasting, travel, and weight concerns influenced consumption patterns. Sharing was minimal and discouraged through counseling. Most participants expressed willingness to continue supplementation, recommending reduced sweetness.

**Conclusion:**

Pregnant women demonstrated high acceptability and adherence to the BEP supplement. Findings support its feasibility for informing intervention design in the main trial.

## Introduction

Pregnant women have higher nutritional requirements that increase their vulnerability to undernutrition and low birth weight offspring [1–3]. In low- and middle-income countries, the prevalence of maternal underweight has remained unacceptably high [4]. Often, inadequate dietary intake and increased physiological demands during pregnancy result in maternal macro- and micronutrient deficiencies, which have a profound impact on poor fetal development and increase the risk of adverse birth outcomes and mortality [5–7].

A possible intervention that may reduce the risk of small for gestational age, low birth weight, stillbirth, and increased birth weight, especially in malnourished women, is the use of prenatal balanced energy protein (BEP) supplements, which are nutritional supplements that provide less than 25% of total energy from protein [8–11]. The World Health Organization (WHO) recommends prenatal BEP supplements in populations with a prevalence of more than 20% of underweight pregnant women (BMI < 18.5 kg/m^2^) [12]. This recommendation is based on evidence from different contexts about the benefits of BEP supplementation during pregnancy [8,13]. Moreover, the Ethiopian Ministry of Health recommends providing BEP supplements to pregnant women with acute malnutrition, defined as mid-upper arm circumference (MUAC) of less than 23 cm, and using ready-to-use therapeutic foods such as Plumpy’Nut and corn-soy blend, until the measurements return to normal [14]. However, implementation of this recommendation is limited due to resource constraints, and the effectiveness of targeted, and potentially most cost-effective strategies needs to be assessed [15].

Despite the recommendation to use nutritional supplements among pregnant women in Ethiopia, several factors hinder their uptake. Cultural and social influences play a significant role, as many recipients prefer traditional foods over introduced supplements due to deeply ingrained dietary practices and taboos [16]. In particular, religious fasting and culturally specific beliefs about food intake during pregnancy may further modify supplement consumption, highlighting the need to examine these practices in relation to adherence. Additionally, the sensory attributes of the supplement, such as its appearance, taste, smell, and texture, can further reduce its acceptance [17]. Tailoring the supplement to the specific needs of pregnant women remains a challenge, as it was initially designed to address malnutrition in children. Limited awareness and inadequate counseling about its benefits further constrain uptake [18].

To better understand the behavioral determinants of BEP supplement uptake, this study draws on the Health Belief Model (HBM). The HBM posits that individuals’ health-related actions are shaped by perceived benefits, perceived barriers, cues to action, and self-efficacy [19]. In the context of pregnancy, women’s decisions to consume BEP supplements may be influenced by their understanding of nutritional risks, beliefs about the supplement’s effectiveness, concerns about taste or side effects, and the presence of supportive counseling or social encouragement. Applying this framework helps structure the exploration of acceptability and adherence, and informs the development of context-sensitive strategies for future implementation.

To inform the design and delivery of BEP supplementation interventions, formative research is needed to understand the factors influencing acceptability, adherence, and sharing practices related to the supplement. This study presents findings from a formative study conducted prior to the initiation of the “Targeting Strategies of Antenatal Balanced Energy and Protein Supplementation in Addis Ababa, Ethiopia” study [15]. The objective of the formative study was to assess the acceptability of, and adherence to, a proposed BEP supplement among pregnant women before the initiation of the main trial.

## Methods

### Study setting

This study was conducted in two of the eleven sub-cities of Addis Ababa, Ethiopia: Akaki-Kaliti and Nifas Silk Lafto. These sub-cities have significant burdens of undernutrition among women of reproductive age. Between 2018 and 2020, among women of reproductive age with a child aged 6–59 months, 17.9% in Akaki-Kaliti and 13.8% in Nifas Silk Lafto had either a low BMI or a low MUAC [15]. Nine of the 14 health centers in the two sub-cities were included in the formative study. These were selected based on their workload, in their ANC and delivery services, and the magnitude of low birth weight.

In Ethiopia, maternal undernutrition is highly prevalent and is a key determinant of poor perinatal outcomes. To address this, national antenatal care (ANC) guidelines recommend ensuring optimal maternal nutrition through appropriate counseling and supplementation. Therefore, MUAC and weight measurements should be taken at every ANC contact to assess nutritional status and take necessary action. A MUAC measurement of less than 23 cm indicates acute malnutrition and signals the need for supplementation with ready-to-use foods [14].

### Study design and population

A qualitative study design was employed to explore participants’ experiences and perceptions of acceptability, while adherence with BEP supplements was assessed quantitatively among pregnant women. The study participants were a purposive sample of pregnant women attending antenatal clinics in Addis Ababa. The selection criteria included 18 years or above, gestational age of up to 24 weeks, willingness to provide written consent, and absence of peanut allergy. Following selection, five eligible women were recruited from each of the nine participating health centers, in coordination with ANC nurses at each facility. The BEP supplement was distributed from January 11–17, 2023. Formative interviews were conducted from February 13–17, 2023. In total, 45 pregnant women received the supplement and were invited to return after one month to share their experience with the study team, including researchers and research assistants. Analysis of participant narratives revealed thematic redundancy across facilities, indicating that saturation had been reached.

Participants received a daily 100-g sachet of Plumpy’Sup®, a ready-to-use lipid-based nutrient supplement commonly used to treat acute malnutrition in children. This product was selected because it is produced and readily available in Ethiopia, and its nutritional composition is optimal for pregnant women. The Ethiopian National ANC Guideline also recommends supplementing ready-to-use therapeutic foods (RUTF) for pregnant mothers with acute malnutrition [14].

Plumpy’Sup® was classified as a balanced energy-protein (BEP) supplement based on alignment with expert recommendations. Each sachet provides 540 kcal, 12 g of protein (9% of energy), and 33 g of lipids (55% of energy), with a broad spectrum of micronutrients. Full compositional details and justification for BEP classification are available in the published protocol [15].

The BEP was distributed to the study’s pregnant women, regardless of their nutritional status, to evaluate their 4-week consumption and adherence to the nutritional supplement. Upon enrolment, all participants received counselling on how to use the supplements, including instructions to consume one sachet per day in addition to their meals/snacks, not to share with others, and to bring both used and unused sachets of the BEP supplements when they came for the in-depth interviews (IDIs) that take place after four weeks of supplement use.

### Data collection

Semi-structured IDIs were conducted with 39 pregnant women who returned to the clinic after four weeks. The interview was conducted at the health facility where pregnant women attend their ANC check-ups. Four female research assistants, trained and with previous experience in qualitative research, conducted the interviews alongside the study researchers. The participants were asked about the supplement’s characteristics, use experience, adherence, and intentions regarding future use. On average, each interview lasts 30 minutes. The interviews were audio-recorded with the participants’ consent. Additionally, detailed field notes were taken, enabling the research team to summarize and highlight key points. These notes served as a valuable resource during the data analysis process, aiding in the identification of emerging themes. All IDIs were conducted in Amharic (the local language), transcribed verbatim, and then translated into English for analysis. The research team used the count of empty sachets and recorded self-reported consumption.

### Data analysis

A framework analysis approach was used to analyze the qualitative data. Researchers and trained research assistants collaboratively conducted the interviews and then coded, categorized, and organized the transcripts using both pre-identified themes and concepts that emerged inductively from participants’ responses. Transcripts were coded using OpenCode software. One researcher performed the initial coding based on the refined framework, and the other researcher independently verified and recoded the transcripts to ensure consistency. Quotations were selected to illustrate key findings and provide depth to participants’ perspectives, with all quotes accompanied by anonymized participant codes to ensure confidentiality. We applied data triangulation by drawing on multiple sources, including collaborative peer debriefing, attention to interviewer-participant dynamics, field reflections throughout all stages of the study, and the use of extensive field notes, which were applied to enhance analytical rigor and minimize bias. Adherence was calculated as the number of sachets reportedly consumed or returned empty by the total number dispensed over the four weeks. Partially used or unopened sachets were considered unconsumed, and missing intake data were treated as non-consumption. Adherence is reported as the proportion of participants consuming ≥80% of sachets and as median [IQR]. To complement this, qualitative interviews explored participants’ experiences with supplement use, and responses related to acceptability were tallied to descriptively summarize common patterns and perceptions.

### Ethical considerations

Ethical approval was obtained from the Addis Continental Institute of Public Health (ACIPH/IRB/008/2022) and Harvard School of Public Health (IRB22–1245) ethical review boards. Written informed consent was obtained from all participants at the time of BEP supplement distribution, covering both the four-week supplementation period and the follow-up interview. Participants were informed about the study’s purpose, procedures, potential risks and benefits, and their right to withdraw at any time without consequence. Interviews were audio-recorded with the participant’s permission, and no financial or material incentives were provided. All personal data was anonymized and stored securely, with identifiable information removed from transcripts to protect participant privacy. Only the research team had access to the recordings and transcripts, which were used solely for research purposes.

## Results

### Characteristics of the study participants

A total of 45 pregnant women were invited to participate in the study and received the supplement. After four weeks, 39 participants were available for interviews, while six did not return for the scheduled follow-up. The reasons for non-return included perceptions of having sufficient food, competing commitments, lack of time, the need to consult with family members, fasting practices, and concerns about child weight gain. Thirty-two participants were between 20 and 30 years old. Twenty participants had attended primary school, and all were either married or living with their partners. Additionally, 20 participants had a family size of three to five members, and 21 identified as Orthodox Christians. Among the 39 participants, 26 were multigravida. Thirty were in their second trimester of pregnancy, and more than half (n = 23) attended their first ANC visit. Supplement consumption over the one month ranged from 0 to 30 sachets, with a mean intake of 21.9 (SD = 9.5). The median number of sachets consumed was 25 (IQR: 18–30). High adherence (≥80% consumption) was observed in 64.1% of participants (95% CI: 48.3–77.9) (Table 1; S1 Table).

**Table 1:** Sociodemographic and Pregnancy-related Characteristics of Participants (n = 39).

Variable	Category	Number of returned participants for interview (n = 39)
Age (years)	<20	2
	20-25	16
	26-30	16
	>30	5
Educational level	No formal education	1
	Grade 1–8	17
	Grade 9–12	13
	Tertiary education	8
Religion	Orthodox Christian	21
	Muslim	8
	Protestant	10
Marital status	Married or living together	39
Total family members	<3	16
	03-May	22
	>5	1
Parity	No child	18
	1 child	14
	2 children	5
	3 children	2
Number of pregnancies	Primigravida	13
	Multigravida	26
Pregnancy trimester	1^st^ (<12 weeks)	9
	2^nd^ (12–24 weeks)	30
Number of ANC consultations	1^st^ contact	23
	2^nd^ contact	14
	3^rd^ contact	2
BEP adherence	Low (<50%)	8 (20.5)
	Moderate (50–80%)	6 (15.4)
	High (≥80%)	25 (64.1)
	(Mean ± SD)	(74.1 ± 32.1)

### Acceptability of the supplement

Of the 39 pregnant women interviewed, 23 (59%) found the supplement’s taste acceptable, often describing it as similar to peanuts or peanut butter. Some participants noted it was overly sweet, which initially made regular consumption challenging, though they eventually adapted. A few reported nausea, dyspepsia, and epigastric discomfort, often attributed to the sweetened nature, prompting suggestions to reduce the sugary flavor. Most participants expressed positive perceptions of the sensory attributes: texture, smell, and color received higher approval, with 32 (82.1%), 35 (89.7%), and 38 (97.4%) participants, respectively, indicating they liked these characteristics. These findings indicate that although taste received mixed responses, other product features were generally well-received. Thirty-three mothers found the supplement easy to consume and use, while six mothers reported difficulty with consumption (Table 2).

**Table 2:** Acceptability of the supplement Based on Sensory Characteristics (n = 39).

Characteristics	Like	Dislike
Taste	23 (59%)	16 (41%)
Texture	32 (82.1%)	7 (17.9%)
Smell	35 (89.7%)	4 (10.3%)
Color	38 (97.4%)	1 (2.6%)
Ease for consumption	33 (84.6%)	6 (15.4%)

*“It smells pleasant, like peanuts. Sometimes, it smells like roasted peanuts.” A105- [Age 19, Parity 1, 2*^*nd*^ *trimester]*

Participants described different methods of intake; some used a spoon to eat from the sachets, others chose to eat it with bread, and some cut the sachets and squeezed the contents directly into their mouths. In contrast, others divided the supplement into smaller portions to eat throughout the day.

Some respondents experienced bloating, nausea, dyspepsia (indigestion), and loss of appetite. Many also felt they drank more water while taking the supplement. Several respondents reported perceived weight gain after starting the supplement; however, these perceptions could not be objectively verified, as weight was not measured before and after supplementation.

*“I experienced vomiting after taking the supplement. However, since you told me it was beneficial, I followed your recommendation and continued taking it.” A405- [Age 30, Parity 1, 2*^*nd*^ *trimester]*

*“It was good that I didn’t drink much before, but now I tend to drink more.” N201- [Age 29, Parity 0, 2*^*nd*^ *trimester]*

### Consumption pattern

Participants reported diverse preferences regarding the timing of supplement consumption. Some preferred taking it mid-morning, around 10:00 a.m., while others preferred intake after lunch or late afternoon, between 5:00 and 6:00 p.m. A few participants consumed the supplement after dinner. Overall, the participants’ timing preferences were diverse.

*“Since I have other children and work responsibilities, morning times (around 10 am and 12 pm) were the most convenient for me to take the supplement.” N301- [Age 24, Parity 1, 2*^*nd*^ *trimester]*

Participants demonstrated a range of supplement consumption patterns. Some began with small portions and gradually increased their intake as symptoms subsided. In contrast, others maintained a consistent consumption pattern throughout the month. These differences in consumption habits were attributed to several factors. Some participants struggled to consume the product due to its tendency to instigate or worsen pregnancy-related symptoms. Others were discouraged by concerns about potential weight gain, which impacted their willingness to take the supplement regularly.

Additional reasons for not adhering to the recommended consumption included the supplement’s milk content, which made it unsuitable for fasting days among Orthodox Christian participants. Conversely, participants who followed the instructions reported perceiving the health benefits of the supplement as valuable for both themselves and their babies.

“*I was taking it every day except for fasting days.” N302- [Age 28, Parity 1, 1*^*st*^ *trimester]*

“*My opinion on the supplement changed when I noticed an increase in energy. Initially, I was uncomfortable using it due to fear of gaining significant body weight, which I intended to monitor. Overall, I found it beneficial.*” *N305- [Age 26, Parity 0, 2*^*nd*^ *trimester]*

Most respondents used the supplement as an addition to their diet rather than a meal replacement. However, a few mentioned occasionally skipping a meal when taking the supplement. For instance, some skipped dinner after consuming the supplement around 5–6 p.m., while others skipped lunch after taking the supplement around noon.

“*No. If I took the supplement, I would not eat any food. I would fill myself up. I usually take it when I’m hungry.* ” *N301- [Age 24, Parity 1, 2*^*nd*^ *trimester]*

The use of the supplement was met with varied responses from family members, such as husbands, in-laws, and neighbors. Many accepted it, believing it would be beneficial for both the woman’s health and the fetus. However, some friends and family members challenged its use due to unfamiliarity with the product or misinformation. One participant faced opposition because neighbors believed the supplement was exclusively for HIV patients.

“*One person has challenged me on taking the supplement, referring to it as something exclusively given to HIV patients.” N305- [Age 26, Parity 0, 2*^*nd*^ *trimester]*

### Supplement adherence and sharing practices

Among the 39 participants, 25 consumed 80% of the supplement, while one did not consume any. Participants reported receiving significant support from various individuals, especially their husbands, to encourage supplement intake, with a particular emphasis on its health benefits for both the mother and the fetus. In some cases, in-laws and neighbors also supported and encouraged its use.

*“My husband always supports me even when I forget and tells me it is good for the fetus’s development.” A202- [Age 23, Parity 0, 2*^*nd*^ *trimester]*

*“My mother-in-law came from a rural area, so she didn’t know much, but she told me that if they gave it to you, you should take it.” A602- [Age 25, Parity 0, 2*^*nd*^ *trimester]*

Participants reported various reasons for consuming less than the recommended amount of the supplement, primarily related to taste and health issues. The overly sweet flavor often led to nausea, sourness, or exacerbated vomiting, causing some to reduce or cease intake. Others cited forgetfulness due to being busy, traveling without the supplement, or skipping doses during fasting periods.

*“It was easy to consume, but when I ate it, it made me throw up and had a sour taste, like burnt coffee. For this reason, I stopped taking it.” A602- [Age 25, Parity 0, 2*^*nd*^ *trimester]*

Participants consistently reported receiving clear guidance from health care providers regarding the intended use of the supplement. Counseling emphasized personal consumption and discouraged sharing with others, including children. However, six participants reported sharing a small amount of the supplement, typically once, with a close family member – most often a husband, sister, son, or daughter; for tasting. These instances were brief, non-routine, and motivated by curiosity. Triangulation with returned sachet counts did not reveal substantial discrepancies. All six women who reported sharing were classified as high adherers based on both self-report and count, suggesting that sharing was limited and had no meaningful impact on adherence. Participants also described strategies to avoid sharing, such as eating privately and explaining to others that the supplement was medicinal. No respondents reported selling or exchanging the supplement.

*“We were told not to give to children or any other person. I haven’t shared for my child.” N205- [Age 21, Parity 0, 2*^*nd*^ *trimester]*

“*She (her daughter) was bothering me, but I fooled her by offering her chocolate and other things. And sometimes I eat it when she is not around and playing with kids, or else if she sees (laugh...) she might bother me*.” *A204- [Age 28, Parity 1, 2*^*nd*^ *trimester]*

### Future use of the supplement

The participants’ willingness to continue using the supplement during pregnancy varied. Twenty-nine expressed willingness to continue, citing its usefulness, taste, perceived health benefits, and disease prevention. However, ten participants reported unwillingness to continue, attributing their decision to discomfort during consumption, religious fasting practices, the supplement’s sweetness, and its soft texture. Some participants noted that their willingness to continue would depend on modifications to the supplement, including reducing sweetness or size, having the option of alternate-day intake, or avoiding use during fasting seasons.

*“No, because the next two months are fasting season. I would have considered it otherwise.” N201- [Age 29, Parity 0, 2*^*nd*^ *trimester]*

*“I don’t think I can take it. Because of nausea and vomiting.” N105- [Age 18, Parity 0, 2*^*nd*^ *trimester]*

*“If it is not sweet, I can take it on alternate days.” A202- [Age 23, Parity 0, 2*^*nd*^ *trimester]*

Most pregnant women emphasized the importance of receiving comprehensive guidance on supplement use, noting that it helped them understand its benefits and the need for exclusive consumption. They highlighted that such education clarified the supplement’s health advantages and reinforced proper adherence. Participants further stressed that explaining the specific benefits for maternal health and fetal development would strengthen correct use. They believed that clear communication from healthcare providers; particularly regarding the supplement’s role in supporting maternal weight gain, promoting fetal growth, and ensuring balanced nutrient intake for both mother and child would enhance adherence.

*“It is wise to advise them not to share. Because if they have children in the household, they are more prone to practicing sharing. Therefore, providing education before distributing the supplement is important.”* N305*- [Age 26, Parity 0, 2*^*nd*^ *trimester]*

*“Because it is beneficial and critical for the baby, I will advise the pregnant woman to take the supplement to help prevent the birth of an underweight child. ” A104- [Age 27, Parity 1, 1*^*st*^ *trimester]*

## Discussion

This formative study assessed the acceptability, adherence, and sharing practice of the BEP supplement among pregnant women in Addis Ababa, Ethiopia. Adherence was high, with 25 of 39 participants (64.1%) consuming at least 80% of the supplement. Most women found the supplement acceptable, particularly its texture, smell, and color.

The BEP supplement demonstrated high acceptability in this study, particularly regarding its sensory properties, such as texture, smell, and color. However, some participants faced challenges with its sweet taste, which was associated with symptoms including nausea and vomiting. Adjusting the sweetness level could improve palatability and support adherence. In Ethiopia, lipid-based BEP products are not commonly used; instead, Corn Soya Bean (CSB) is the food supplement distributed by the Ethiopian Ministry of Health and non-government organizations (NGOs) [20]. Evidence from studies in other countries has shown that lipid-based supplements are often highly rated for their overall taste, color, and smell [1].

Participants exhibited diverse consumption patterns, influenced by personal schedules and health responses. Most consumed the supplement after meals, with preferences varying between breakfast, lunch, and the afternoon. These preferences may reflect cultural eating patterns, including typical meal timing, social norms around food intake, and beliefs about appropriate foods during pregnancy. Several studies indicate that pregnant women prefer particular times for consumption based on when pregnancy symptoms subside [11,21]. Despite initial reluctance or adverse effects such as bloating or nausea, many participants adapted to its consumption. These findings emphasize the importance of personalized counseling to manage expectations and mitigate discomfort during initial use [18,22].

Counseling from health workers, who emphasized exclusive use and discouraged sharing, was consistently cited as critical for maintaining supplement use. Participants’ understanding of the supplement’s purpose and its framing as “medicine” helped them resist social pressure to share. Family support further strengthened adherence, with husbands and relatives encouraging consumption, particularly when benefits for maternal and fetal health were highlighted.

Barriers included taste-related nausea, fasting practices, and forgetfulness. Nonetheless, most pregnant women recognized the supplement was intended for their personal use and were reluctant to share. Health education and clear communication during distribution were essential for reinforcing exclusive use, consistent with the previous research [9]. Approaches such as counseling and counting returned sachets may help reduce sharing [2]. Family members also discouraged sharing, thereby reinforcing adherence [23]. These findings underscore the importance of healthcare providers clearly communicating the benefits of the supplement, providing practical instructions, and discouraging sharing to improve adherence [9,24,25].

This study had several strengths. IDIs were conducted immediately after the 4-week supplementation period to minimize recall bias. Additionally, sachet counting helped reduce underreporting or overreporting. However, the study also had limitations. Acceptability, adherence, and sharing experiences were assessed through self-reporting, which may have introduced social desirability bias. Participants may have provided responses they perceived as socially acceptable, potentially underreporting unfavorable views about the BEP supplements. Although sachet counts and timely interviews supported participant responses, the absence of observational triangulation or random spot checks may have limited validation.

## Supporting information

S1 Table Patterns of Supplement Adherence Stratified by Educational Level and Parity among Study Participants. (DOCX)
