# Supplementary material for: Acceptability and adherence of Balanced Energy Protein (BEP) supplementation among pregnant women in Addis Ababa, Ethiopia: A Formative Study
Source: PLoS One. 2026 Mar 6;21(3):e0344089. doi: 10.1371/journal.pone.0344089 (PMC12965526; doi:10.1371/journal.pone.0344089)
Supplement: S1 Table — (DOCX) [file pone.0344089.s001.docx]

**Supplementary Table 1: Patterns of Supplement Adherence Stratified by Educational Level and Parity Among Study Participants**

| **Variables** | **High Adherence** | **Moderate Adherence** | **Low Adherence** | **Total (n=39)** |
| --- | --- | --- | --- | --- |
| **Educational Level** |  |  |  |  |
| No/Primary education | 9 | 3 | 6 | 18 |
| Secondary education | 11 | 1 | 1 | 13 |
| Tertiary education | 5 | 2 | 1 | 8 |
| **Parity** |  |  |  |  |
| 0 | 12 | 4 | 2 | 18 |
| 1 | 9 | 2 | 3 | 14 |
| 2 | 2 | 0 | 3 | 5 |
| 3 | 2 | 0 | 0 | 2 |
